# Large-Area and Low-Cost Force/Tactile Capacitive Sensor for Soft Robotic Applications

**DOI:** 10.3390/s22114083

**Published:** 2022-05-27

**Authors:** Amir Pagoli, Frédéric Chapelle, Juan-Antonio Corrales-Ramon, Youcef Mezouar, Yuri Lapusta

**Affiliations:** 1 Institut Pascal, Université Clermont Auvergne, Clermont Auvergne INP, CNRS, 63000 Clermont-Ferrand, France; frederic.chapelle@sigma-clermont.fr (F.C.); youcef.mezouar@sigma-clermont.fr (Y.M.); yuri.lapusta@sigma-clermont.fr (Y.L.); 2 CiTIUS (Centro Singular de Investigación en Tecnoloxías Intelixentes), Universidade de Santiago de Compostela, 15782 Santiago de Compostela, Spain; juanantonio.corrales@usc.es

**Keywords:** soft sensor, tactile sensor, capacitive sensor, calibration, neural network, soft robot, soft pneumatic actuator

## Abstract

This paper presents a novel design and development of a low-cost and multi-touch sensor based on capacitive variations. This new sensor is very flexible and easy to fabricate, making it an appropriate choice for soft robot applications. Materials (conductive ink, silicone, and control boards) used in this sensor are inexpensive and easily found in the market. The proposed sensor is made of a wafer of different layers, silicone layers with electrically conductive ink, and a pressure-sensitive conductive paper sheet. Previous approaches like e-skin can measure the contact point or pressure of conductive objects like the human body or finger, while the proposed design enables the sensor to detect the object’s contact point and the applied force without considering the material conductivity of the object. The sensor can detect five multi-touch points at the same time. A neural network architecture is used to calibrate the applied force with acceptable accuracy in the presence of noise, variation in gains, and non-linearity. The force measured in real time by a commercial precise force sensor (ATI) is mapped with the produced voltage obtained by changing the layers’ capacitance between two electrode layers. Finally, the soft robot gripper embedding the suggested tactile sensor is utilized to grasp an object with position and force feedback signals.

## 1. Introduction

Inspired by nature, scientists have tried to build a new field of robotics influenced by human body interactions called soft robotics. Thanks to recent advances in smart and soft materials, the new types of soft actuators can perform different complex tasks. They have several advantages, including infinite degrees of freedom (DOFs) and lightweight, easy, and cost-effective fabrication. Unlike conventional robots, soft robots utilize various types of actuation, such as pressurized fluids, electric or magnetic fields, temperature, and chemical reactions [1]. This specificity increases the variety of soft robot applications in different areas, including manipulation [2], grasping [3], locomotion [4], medical applications [5], and underwater robot [6] applications. Although their deformable features enable them to be used in uncontrolled environments without requiring complex protection or stability control algorithms as in hard robots, their morphological structures restrict utilizing traditional sensors such as encoders, metal or semiconductor strain gauges, or inertial measurement units (IMUs) [7]. In magnetic sensors, the stiffness of the soft actuator is changed by adding the magnet and the Hall element [8], and optoelectronic sensors require a transparent material for transmitting light [9], but resistive or capacitive sensors are the most commonly used method due to fewer limitations in measuring force, curvature, or tactile sensing. Elastomer sensors allow a minimal impact on robot actuation. On the other hand, soft robots’ sensing design should be at least flexible or ideally stretchable. Moreover, the integrated sensors should not increase the stiffness of the soft actuator. Recent embedded sensor advances and applications in soft actuators in terms of performance, resolution, stretchability, self-healing, and self-powered capability are reviewed in [10,11]. Tactile sensors are generally made of lightweight, stretchable, and elastic materials. Li et al. reviewed the last developed sensing technologies in soft robotic systems, including resistive, capacitive, optoelectronic, and magnetic sensors [12]. Wang et al. [13] reviewed the advances and potential challenges in soft robotics sensing. Therefore, flexible and curvature sensors are still interesting subjects for closed-loop control of soft actuators. In a resistive sensor, applying mechanical pressure changes the strain and, consequently, the conductivity. In a capacitive sensor, the conductivity is dependent on the geometry area of the dielectric materials between two electrodes [14]. Koivikko et al. integrated resistive sensors in a soft gripper to detect the curvature [15]. Yang et al. [16] used a thin layer of paper as electrodes, which was printed using resistive and capacitive nano-silver ink. The proposed sensor was able to detect the bending angle and the object’s proximity. Most electrode materials embedded in soft grippers as capacitive sensors are made of conductive particles of carbon black (CB) [17], conductive ink [18], graphene [19], liquid metal [20], and carbon nanotubes [21]. Other types of materials are reviewed in [22] that can be operated as electrodes in flexible sensors. Gafford et al. used a rapid prototyping method, namely shape deposition manufacturing (SDM), to fabricate a surgical three-finger-gripper with an embedded microelectromechanical pressure sensor on its fingertips [23]. Cheng et al. [24] implemented a large-area, highly-twistable, artificial skin by winding the copper wires around an elastic nylon line. The applied force and tactile sensing can be detected through electrical resistance and pressure, respectively. Ho et al. [25] developed elastomer fingers with a multi-layer fabric capacitive sensor to detect proximity and contact feedback information, and to grasp delicate objects. A highly stretchable tactile capacitive sensor for a soft pneumatic actuator is proposed in [26]. Lee et al. [27] presented a flexible capacitive 8 × 8 sensor array. The sensor structure consists of parallel plate capacitors that simultaneously enable sensing normal and shear directions. The copper electrodes are patterned on the PDMS substrate. By applying an external force, the air gaps are deformed, leading to a change in capacitance. A flexible polymer-based, three-axial, capacitive sensor was developed by Brzynska et al. [28]. They integrated three kinds of polymers with standard metallization in a cleanroom batch-type fabrication process. This sensor can be used as a promising candidate for artificial skin applications. A capacitive pressure sensor with polyvinylidene fluoride (PVDF) was proposed in [29]. The pattern transformation of anodized aluminum oxide is utilized for fast, large-scale sensor fabrication. However, most of the mentioned methods required complex and expensive fabrication techniques, while the main important goal of soft robot systems is simplifying the robot mechanisms, including mechanical and electrical components without reducing the robot’s functionality. The 3D-printing method is employed to integrate hydrogel electrodes into the silicone. Due to better performance, easier implementation, and calibration compared to resistive soft sensors, capacitive sensors are widely used in tactile sensors. Furthermore, they can also detect multi-touch gestures and allow for the inferring of information. Due to these advantages, capacitive sensing was selected in this study for soft robot applications.

**Figure 1:**
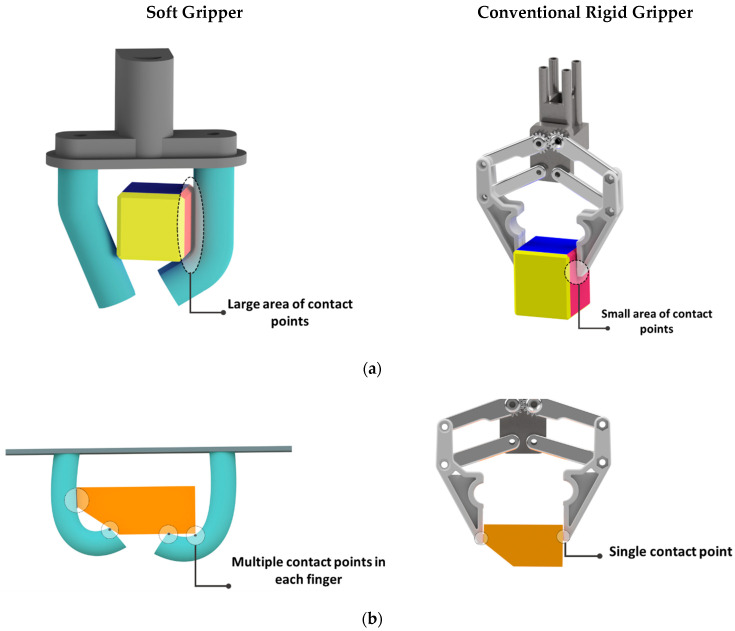
Comparison of the grasping performance of a soft and a conventional rigid gripper. Soft grippers have advantages including (**a**) large-area contact points and (**b**) multi-touch contact points at the same time.

In this work, a new multi-touch, large-area, capacitive sensor is proposed. Our proposed sensor exhibits several advantages, such as stretchability, fast response, and low-cost materials for measuring contact points and applied forces on soft grippers. Compared to conventional grippers, soft grippers can grasp an object with a larger contact area, which consequently requires covering a wide range of sensing regions with high spatial resolution. Figure 1 compares the grasping technique of a conventional rigid gripper with a soft gripper. The rigid gripper generally grasps an object with the tipping point of each finger. The force or tactile sensor can be embedded at the fingertip to cover this area. For this purpose, many commercial sensors with small dimensions are suggested in the literature, including the Hall effect sensor [30], tactile sensor [31], and force-resistive sensor [32]. While in the soft gripper, as shown in Figure 1a, large areas are used for obtaining the object, and the sensor should be able to cover this area. Many soft, flexible, and stretchable sensors for soft grippers have been proposed, but most of them focused only on the tipping point of the gripper, which is in contrast with the nature of the application of the soft grippers. For instance, Cho et al. [33] developed an EGaIn tactile sensor to measure forces at the end of the finger. Then, the sensing response experiment confirmed the performance of the object’s grasping state. In a similar work, Hao et al. [34] developed an EGaIn tactile sensor at the fingertip of the gripper to identify objects with sensory feedback. Therefore, it is necessary to develop a large-area sensor specifically according to the soft gripper dimension. Moreover, unlike a rigid gripper, the object can have multiple contact points with each soft finger, as shown in Figure 1b. Hence, the sensor should be capable of measuring multiple points simultaneously. Although most capacitive sensors like e-skin sensors are highly stretchable and can detect multiple contact points, they are just sensitive to conductive objects (e.g., the human body) [35], while the behavior of the proposed sensor is independent from the object material.

In the following study, a neural network is used to calibrate the applied forces to achieve higher accuracy. Then, the calibrated sensor is embedded into a soft finger to validate the grasping of an object by sending out the contact position and related force as a feedback signal. Sensor calibration includes a non-linear process. Recently, artificial neural networks (ANN) have been used for modeling non-linear systems. They can solve highly complex problems on mathematical calculations or other classical procedures without needing to explicitly define the model structure [36]. They reduce the modeling process to network training, which is useful, especially for non-linear sensor calibrations when sensor array signals are used to calculate the parameters [37]. In the literature, ANN-based soft sensors are usually employed to find the relationship between inputs and outputs by minimizing the mean square error. After calibrating the sensors, the trained model can predict the output whenever required. One drawback of ANNs is that the training time is long. Almassri et al. [38] proposed the Levenberg–Marquardt back-propagation artificial neural network (LMBP-ANN) model for self-calibrating a pressure sensor for reliable grasping by wearable robotic hand gloves. The model successfully predicted the pressure in the presence of hysteresis, creep, and nonlinearity. The back-propagation (BP) neural network was suggested by Ye et al. [39] for self-calibration of the non-array tactile sensor’s structure. This design does not require arrays of electrodes; therefore, it is easy to fabricate and covers a large force detection area. The rest of this paper is organized as follows: The following section presents the conceptual and operating principles of the proposed sensor. Then we discuss the manufacturing procedure and tactile performance of the sensor. After that, an application of the designed sensor in a soft gripper is introduced. Finally, a conclusion and future work are reported.

## 2. Methods

**Figure 2:**
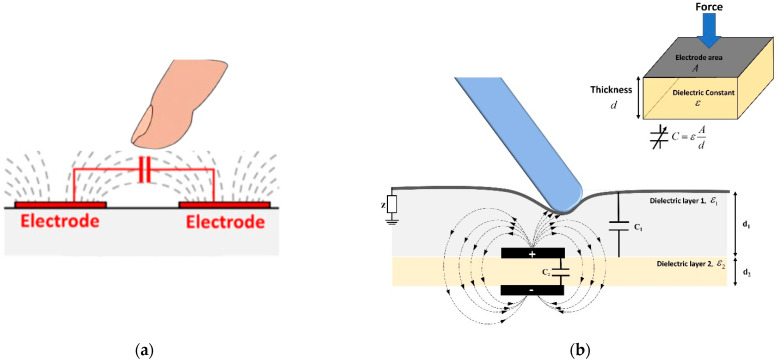
Schematic view of the working principles for the (**a**) typical capacitive sensor and (**b**) proposed sensor.

**Figure 3:**
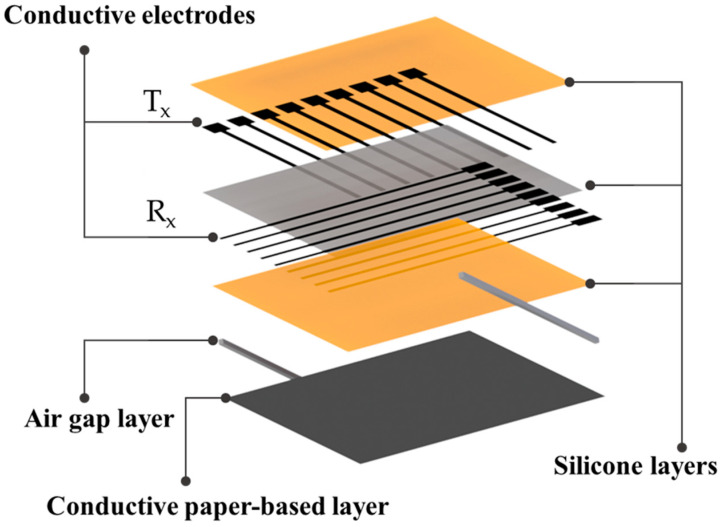
Schematic illustration of the proposed sensor’s internal layers.

Capacitive sensors mainly consist of two conductive layers separated by a dielectric elastomer layer. The capacitance changes when the object moves nearer to the electrodes (Figure 2a). This also changes the local electric field. In the most recent approaches, the object should be conductive or semiconductive with significant impedance for observable changes in the electric field. However, some approaches depend on the sensitivity material of electrodes, such as an elastic carbon nanotube (CNT). In this case, the capacitance is also altered by non-conductive materials [21]. Our work aims to develop a new type of capacitance sensor that can measure contact points by applying pressure with any object without requiring conductive materials. The schematic view of the working principle of the proposed sensor is presented in Figure 2b. The contact point can be detected by changing the capacitance of the touching point area. It consists of two orthogonal arrays of electrodes: vertical lines (T_x_) for sending, and horizontal lines (R_x_) for receiving. A small voltage is applied to T_x_ to build an electrical field between the electrodes. The displacement current resulting from changing the electric field is measured at R_x_. A conductive flexible substrate with a ground connection is designed at the top of the layers, as described in Figure 3. Bringing the object closer to the surface drains a certain amount of field lines between T_x_ and R_x_, which can be observed to specify the touchpoints. Furthermore, the other complementary effect of this design type is pressure sensitivity. By applying an external force, the electrode distance changes, as shown in Figure 2b. The amount of force can be measured from the produced current displacement. The capacitance for a parallel plate can be described as calculated by Equation (1),

(1) $$C_{sensor}=\varepsilon \;\frac{A}{d}=k\;\varepsilon_{0}\;\frac{A}{d}$$

where *A* represents the electrode area, *d* represents the dielectric thickness, $\varepsilon_{0}$ is the permittivity of the vacuum, and k is called the dielectric constant of the layer between two plates. The capacitance can be varied by changing the thickness of the dielectric layer between two plates. Our sensor is composed of two capacitors that are connected in parallel. The total capacitance is calculated as

(2) $$C_{T}=C_{1}+C_{2}$$

with

(3) $$C_{1}={k_{1}\;\varepsilon }_{0}\;\;\frac{A_{1}}{d_{1}},\;C_{2}=k_{2}{\;\varepsilon }_{0}\;\;\frac{A_{2}}{d_{2}}$$

we obtain

(4) $$C_{T}=\varepsilon_{0}\;(k_{1}\frac{A_{1}}{d_{1}}+k_{2}\frac{A_{2}}{d_{2}})$$

where $\varepsilon_{0}$ equals $8.854\times 10^{-12}\;F/m$ and $k_{1}$ for air is considered as 1 F/m, while for the Ecoflex 00-50, this constant is around $k_{2}=2.65$ F/m [40].

## 3. Materials and Fabrication of the Flexible Capacitive Sensor

**Figure 4:**
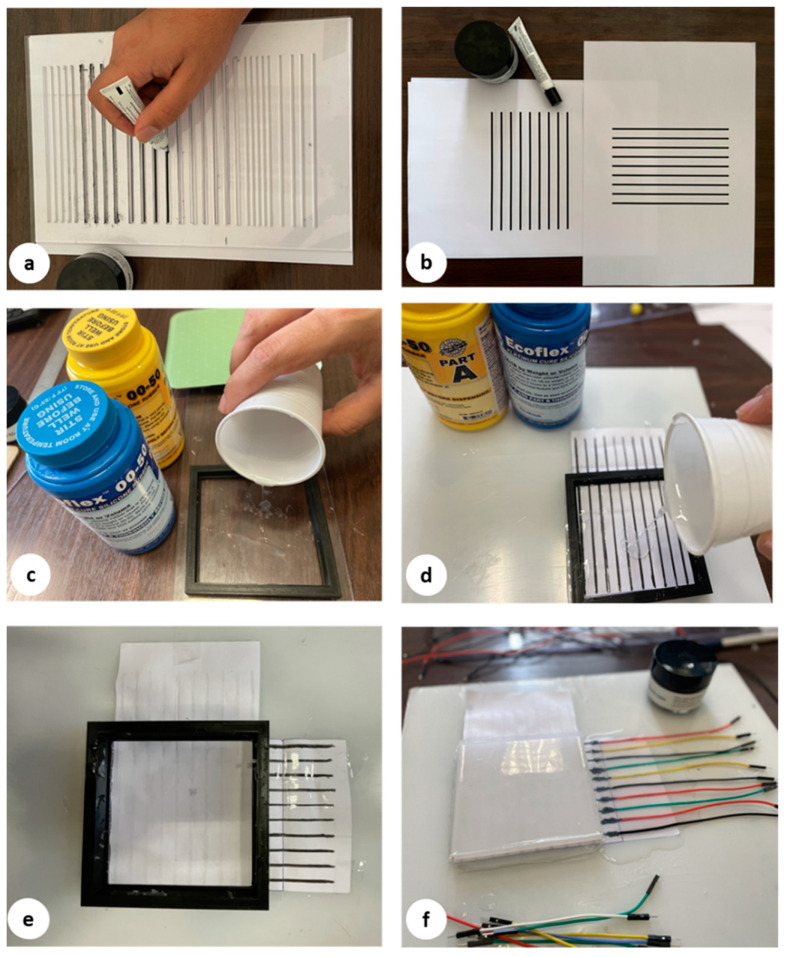
(**a**–**f**): Manufacturing procedure of the proposed sensor.

Figure 4 presents the fabrication procedures of our flexible capacitive sensor. The sensor architecture was developed with a top layer of silicone, two conductive layers for horizontal and vertical tracks, two layers of silicone elastomer, and a conductive shield as a bottom layer. It should be fabricated layer by layer. Due to its prominent features, including lightweight, hyper elasticity, and fast and easy fabrication, silicone is one of the most widespread materials used in soft robotic systems. Ecoflex is one of the popular silicones frequently used. It is commercialized by Smooth-On [41]. Considering the application, the shore hardness range of silicone can be selected from 00-10 to 00-50. In this article, the silicone Ecoflex 00-50 was used to fabricate the sensor layers. The elastomer material properties are summarized in Table 1. These properties allow Ecoflex to expand from its primary dimension many times without tearing, making it a proper choice for soft sensor applications. For more information about other types of silicone used in soft robot applications and their mechanical properties, the reader is referred to the review article [42]. The conventional molding technique was used to fabricate different layers of the proposed sensor. A 3D printer was used to make the layer frames with different thicknesses to find the optimal thickness of the layers related to the sensor’s sensitivity. Ecoflex consists of two parts that should be mixed well with the same ratio, according to the manufacturer’s instructions. After mixing the two silicone parts and before pouring them into the mold, vacuum degassing was applied for around three minutes to remove air bubbles. Ecoflex layers were cured after three hours at room temperature. The curing time can be less than an hour by utilizing an oven to heat the mixing liquid up to a temperature of around 70°. Table 2 compares the materials cost of the proposed sensor with the most used materials to fabricate tactile/capacitance sensors. As shown in this table, the total price of our proposed sensor is noticeably lower than the other methods. 

**Figure 5:**
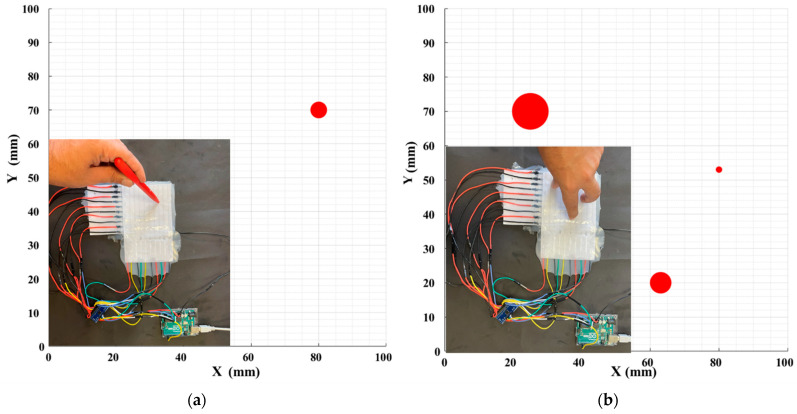
Multi-touch force/tactile capacitive 100 × 100 mm soft rectangular pad: (**a**) non-conductive object (plastique pen) and (**b**) conductive object (human finger) with different pressures applied.

**Figure 6:**
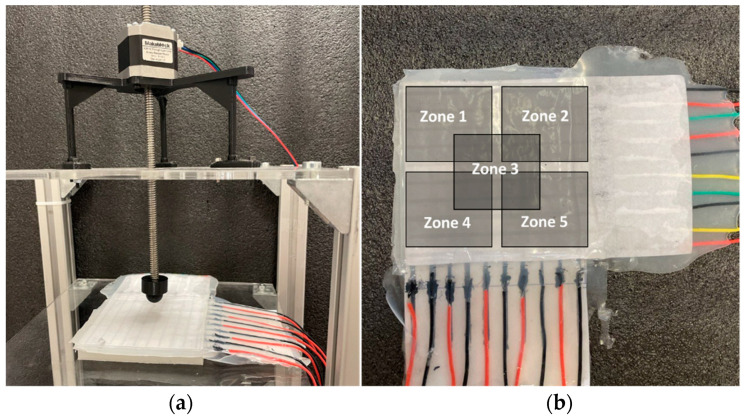
Setup for operating different tactile tests: (**a**) Attaching different probe sizes to the stepper motor for applying normal force and (**b**) dividing the sensor into five test zones to investigate the sensor performance.

**Figure 7:**
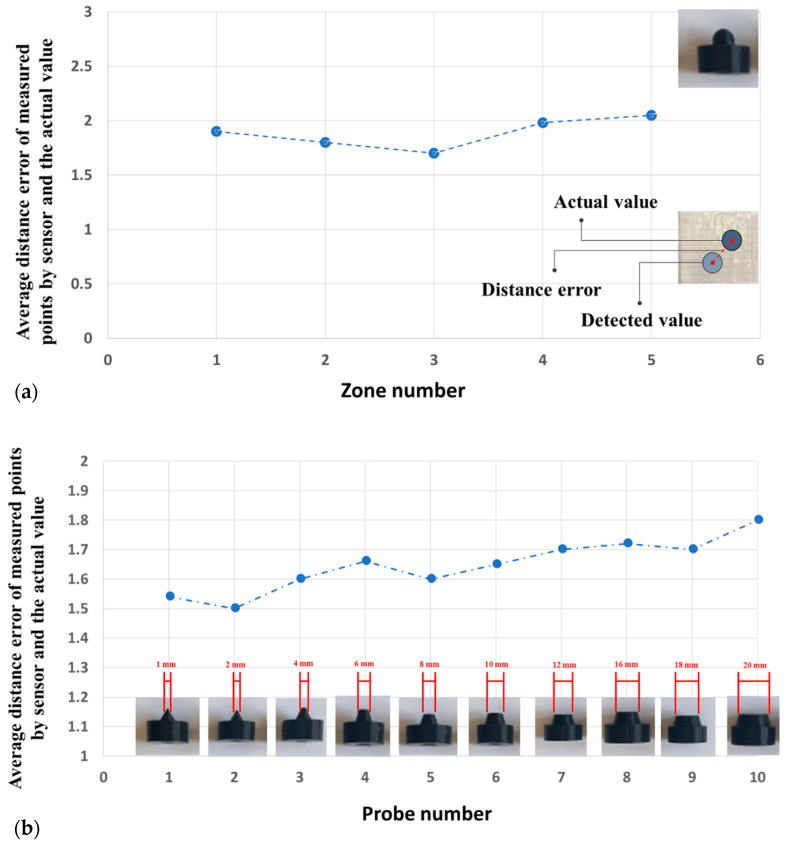
The average distance error of measured point by the sensor and actual value: (**a**) Each probe is touching Zone 1 to Zone 5, repeating 10 times to record the sensor measuring point, and (**b**) different probes are utilized to touch the center of the sensor 10 times.

The fabrication process starts by pouring Ecoflex (Figure 4c) with 2 mm as the base substrate. After curing the top layer consisting of Ecoflex 00-50 with a thickness of 3 mm, the painted paper, including 9 horizontal electrodes, is placed on the top layer. The distance between these electrodes is set to 10 mm (Figure 4b). Then, these electrodes are covered by a very thin layer of silicone (Figure 4d), which affects the measured range of pressures according to Equation (1). To achieve maximum sensitivity, different manufacturable silicone layers (0.2 to 1 mm) were tested to find the largest variance of the forces. Each layer was tested by applying normal force produced by a stepper motor, and then measuring the output signal. The thickness of silicone less than 0.5 mm shows a wide range of signal change outputs, which is desirable for the proposed sensor. In the next step, the second layer of electrodes is laid down perpendicularly compared to the previous electrode layer to build a 9 × 9 electrode matrix grid (Figure 4e). Finally, the conductive paper shield covered by silicone is attached to the electrode layer with an air gap. To find the appropriate value of the air gap between the electrode layer and the conductive shield, ensuring maximum sensitivity, different distances between 1 mm to 5 mm were tested. The optimal air gap was found between 2 and 3 mm. An air gap lower than 2 mm increases the shortcut circuit and saturation possibility, and an air gap bigger than 3 mm reduces the sensitivity by reducing the variance of output signal changes. To easily make the prototype samples, the water-based, non-toxic Bare Conductive electric paint, namely Bare Conductive [45], was chosen for electrodes and the conductive shield, which is provided by the manufacturer in a 10 mL tube. The electric paint dries at room temperature and is used to draw the electrodes. The resistivity range of these materials varies between 33–55 Ω/m [46]. It contains conductive carbon, water, and natural resin. Therefore, it can be solved easily in water. To have a unified electrode size, the conductive ink is patterned on a filter glass with a 2 mm thickness (Figure 4a). The electrodes are then connected with wires (Figure 4f) to the hardware-sensing platform by a Muca breakout. This data acquisition system was presented by Tesseyer et al. in [47]. The FT5316DME controller in this breakout provides 33 connectors (maximum 12 sensing electrodes and 21 transmitting electrodes). This sensor can detect 5 multi-touch coordinates at the same time and send them out via i2C to the Arduino Uno. A serial communication then transports the data from the Arduino to a PC. The external touch position can be calculated by reading the row and column data separately, which represent the X and Y coordinates, respectively. The MATLAB software is utilized for communicating with the microcontroller board to receive, log, visualize, and analyze the external contacts in real time. The measurement results in the 100 × 100 mm soft rectangular pad and mutual-capacitive readout are represented in Figure 5. When the object is touching the surface of the pad, the x, y coordinates and magnitude of contact force are calculated and depicted in real time. Two types of experiments, a non-conductive object (plastic pen) and a conductive object (human finger), are tested to show the sensor’s performance. As shown in Figure 5, the sensor can detect three touchpoints with different pressure amounts simultaneously. The circle radius shows the capacitance changes of the touching pad. By increasing the pressure, circle size will be increased. For instance, we applied more pressure with our thumb finger. To reduce the background noises, small changes in capacitance (less than 5%) were filtered and are not presented in these pictures. Figure 6 shows the designed experimental setup to evaluate the impact of the touch durability and the consistency of the proposed sensor. The sensor is divided into five zones. In each zone, the same position is touched 10 times with a five-second delay. A stepper motor controls the contact speed and applies a constant force for each touch. Then, the sensor’s average distance error between the measured and actual position of the contact point value is calculated. This procedure is repeated 10 times, and average amounts are plotted in Figure 7a. The results show that the sensor performs better in the center zone area 3. In the second experimental test, 10 probes of different sizes are used to assess the sensitivity of the sensor. A 3D printer is used to fabricate 10 probes with a range size between 1 mm to 20 mm. Each probe touches the point center of the sensor 10 times, and the average distance error is calculated and depicted in Figure 7b. The results show that the sensor can detect the contact point of different probe sizes with good accuracy even after 100 touches. The sensor shows better precision for sharp objects. One potential challenge of using silicone as a substrate of the sensor is during cyclic loading. Hysteresis due to the nonlinear viscoelastic behavior of silicone can be observed, especially when large deformation occurs. Some research has been done in this field, focusing on fiber-reinforced elastomers to reduce the effect of cyclic loading and hysteresis [48]. In our sensor, we used paper for electrodes and a top layer of silicone, which acts as a fiber reinforcement, increasing the durability of the sensor. Moreover, in this case, the deformation of the sensor is small. Therefore, in this study, the hysteresis effects can be neglected.

## 4. Calibration Procedure for Soft Robot Applications

**Figure 8:**
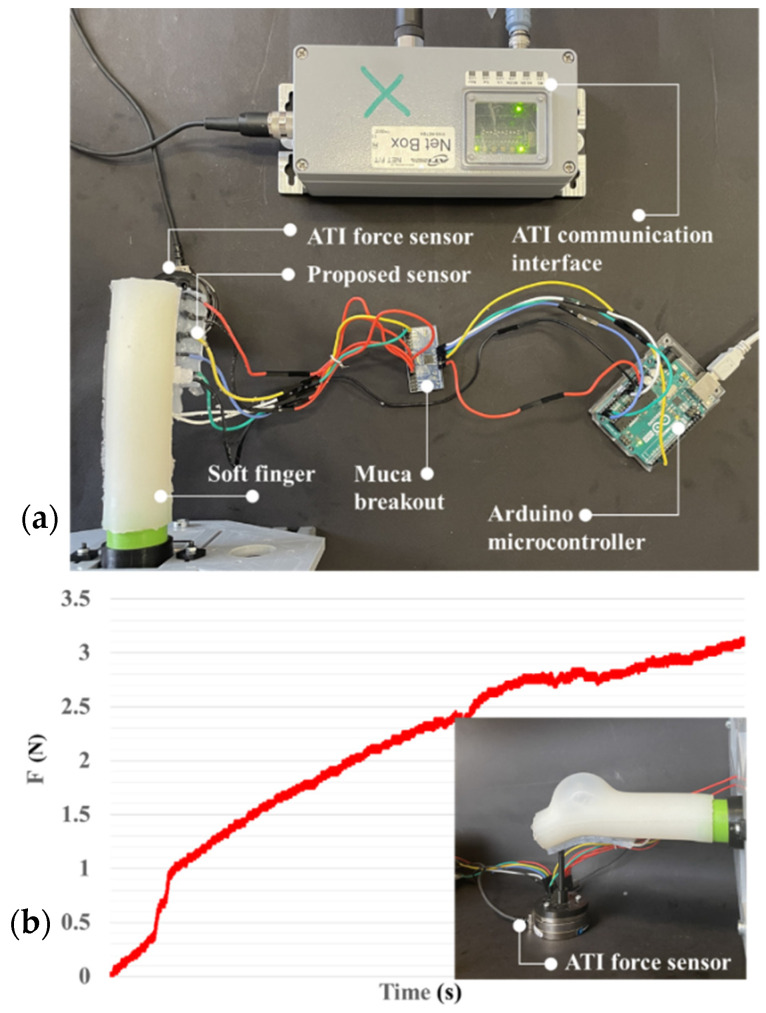
(**a**) Calibration setup assembly and (**b**) testbench for measuring the finger’s force.

**Figure 9:**
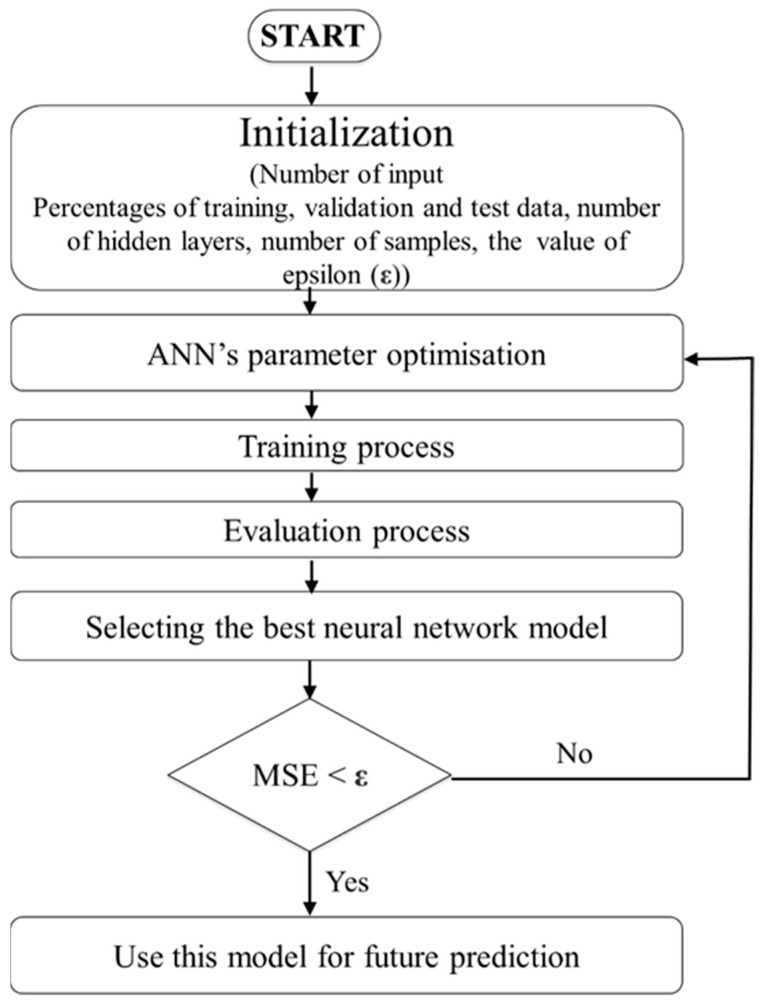
Artificial neural network flowchart for calibrating the proposed sensor.

**Figure 10:**
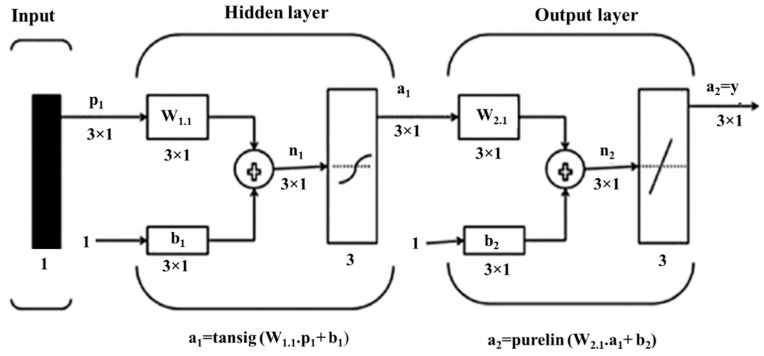
The proposed two-layer feedforward network to calibrate the soft sensor.

**Figure 11:**
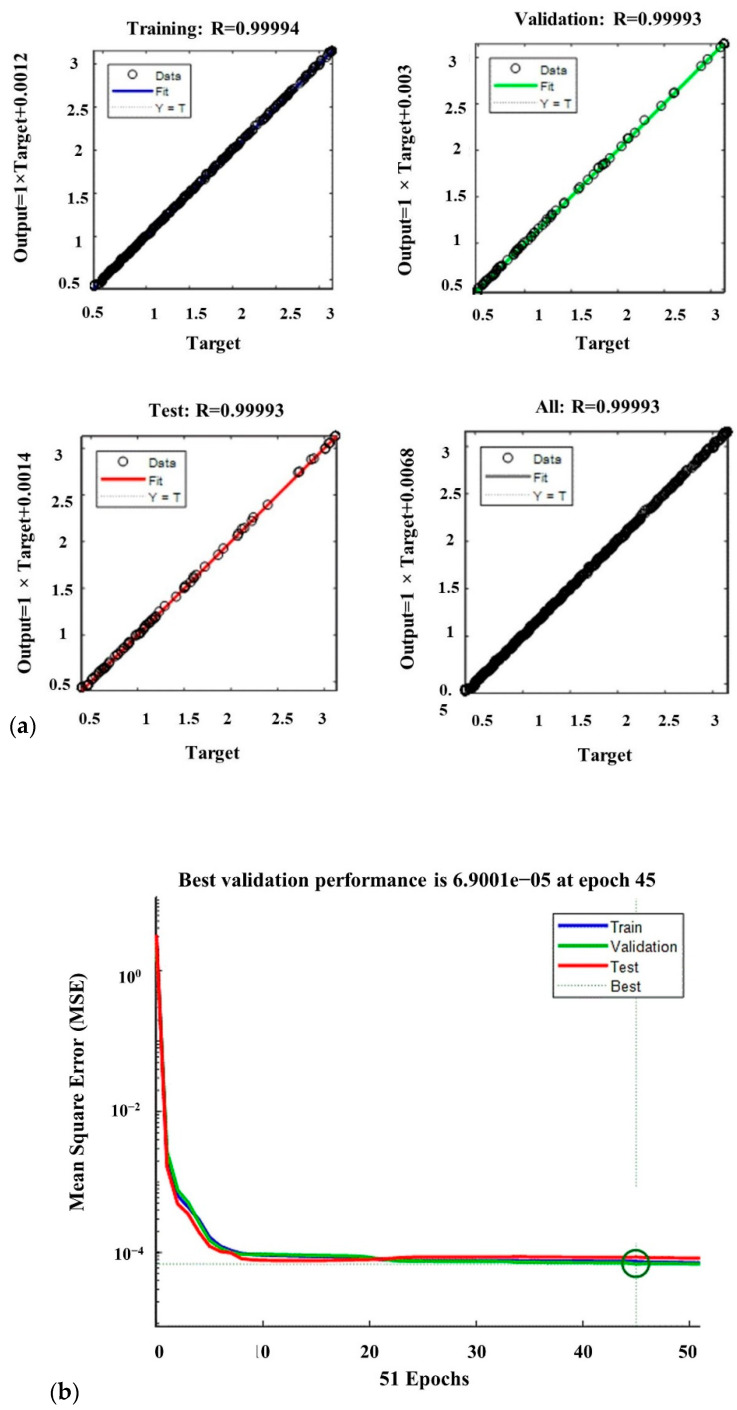
(**a**) Approximation capability of the trained neural network and (**b**) mean squared error of the finger’s force.

In our previous works, we developed a soft robotic finger with a movable joint for enhancing the shape control of soft actuators [49]. Later, we proposed a soft robotic gripper with three fingers for in-hand manipulation [50]. An open-loop control law was applied to control the pressure. The installation of the proposed tactile sensor on the fingers of this gripper can increase its grasping quality by using its data as feedback for the fingers’ control. The fabrication of the sensor here is composed of five horizontal lines and two vertical lines (5 × 2) to gather the sensing data as in the previous section regarding the surface dimension of the finger (50 × 25 mm). As shown in Figure 8, the sensor can be attached easily to the finger by pouring a very thin layer of silicone between the sensor and the soft finger. After curing the silicone, the sensor and finger are unified. The finger was used to push on the ATI FT14000 sensor, and the produced voltage corresponding to the applied force was measured by the ATI sensor. The maximum force that the finger can apply was measured by the ATI sensor and was around 3.2 N. To produce this force range, a small pump with a working pressure of around 14 kPa was used. Due to the background crosstalk, finding the proper equation between the force and voltage is very difficult. Artificial neural networks are a familiar way to model the behavior of unknown systems in different areas such as robotics, manufacturing, and optimization. Several studies have been conducted on the application of ANNs to model and forecast various applications because of the ANN’s ability in modeling complex relationships between inputs and outputs or finding data patterns. ANN can be described as a group of simple processing elements called neurons. Neurons aim to find a mapping between the input space (input layer) and the desired space (output layer) by identifying the relationship between their data. Each hidden layer is responsible for transforming the propagated data to the next layer. The learning process continues for several iterations until the average mean square error (MSE) attains an asymptotic value. Figure 9 represents the flowchart of the ANN development. The process used for network training is called a learning algorithm, which is designed to change the junction weights of the network to obtain the desired objectives. The ANN in this study consists of a two-layer feedforward network with a tangent sigmoid transfer function (tansig) between the input and hidden layer, a linear transfer function (purelin) between the hidden and output layer, and Levenberg–Marquardt back-propagation training due to its fast convergence compared to alternative back-propagation methods. A sample of a two-layer feedforward network is illustrated in Figure 10. These networks include input, hidden, and output layers, where the hidden layer neurons’ number is determined by an experimental design and analytical method. Five hundred experiments were executed. The data are randomly divided into three training, validation, and testing subsets to avoid any bias (70% for training, 15% for validation, and 15% for testing). Therefore, 350, 75, and 75 samples were used for training, validation, and testing subsets, respectively. Many methods are available in the literature to determine the number of hidden layer neurons. In this research, the formula proposed by Hecht–Nielsen [51] was used to specify the number of neurons in the hidden layer. One of the best predictions for the number of neurons in the hidden layer is as follows:

(5) m=2n+1,

where *m* represents the number of neurons in the hidden layer and *n* is the number of input neurons. Considering that there is one input, the number of hidden layer neurons is 3. To compare the optimization algorithms, first, it is necessary to design an ANN, and then evaluate the performance of the ANN in predicting the objective function value. Indeed, it is essential to measure how well the ANN adapts to the training data. It is observed that if the ANN generalizes well, it has captured the system characteristics. Some different performance measures are used through the training process to evaluate ANN architectures. In this study, the mean square error (MSE) and determination coefficient (*R*^2^) are considered as the performance function. The mean square error (Equation (6)) is used to determine how well the ANN output fits the desired output presented in the training data. Therefore, the determination coefficient (Equation (7)) is related to the difference between the network output and the desired output [52].

(6) $$MSE=\frac{1}{N}{\sum_{i=1}^{N}\left(y_{prd,i}-y_{\exp,i}\right)}^{2}$$

(7) $$R^{2}=1-\frac{\sum_{i=1}^{N}\left(|y_{prd,i}-y_{\exp,i}|\right)}{\sum_{i=1}^{N}\left(y_{prd,i}-y_{m}\right)}$$

where *y_prd,i_* represents the predicted value of the objective function using the ANN model, *y_exp,i_* is the experimental value of the objective function, *N* shows the number of data, and *y_m_* represents the average of the experimental value of the objective function. Figure 11a plots the values of the ANN model plotted versus the corresponding experimental values to visualize the modeling capabilities of the ANN models. The *R*^2^ for the training, validation, and testing datasets are 0.99994, 0.99993, and 0.99993, respectively. 

**Figure 12:**
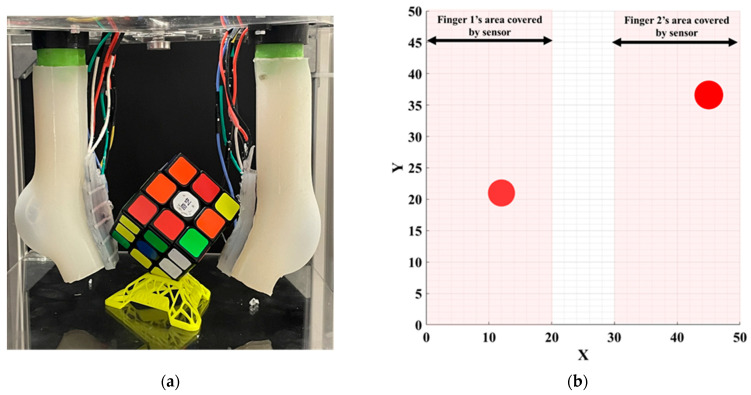
(**a**) Two calibrated capacitive/tactile sensors used for a soft robotic grasping application. (**b**) Both sensors can accurately measure the contact point and applied force (2.5 N).

The high values of *R*^2^ show that the trained ANN model is capable of finding the relationships between the decision variables and the objective function with high accuracy. Consequently, the designed neural network is sufficiently efficient to predict the values of the objective function. The ANN converged very fast to the desired accuracy. Figure 11b reports the average mean square error (MSE) for 51 runs. At 45 epochs, the value of the MSE is 6.9001 × 10^−5^, which is the best performance. Table 3 shows the specifications and parameter values that are used in the LMBP-ANN model. To evaluate the applicability of the proposed sensor, we carried out experiments of the calibrated sensor assembled with a soft gripper (Figure 12a). The soft gripper consists of two fingers to grasp the object. Figure 12 shows that the applied force on several contact points can be detected with a good approximation in the task of grasping a cube. The captured data have been smoothed by calculating the moving average values over ten sensing data frames. The measured force limit can be increased by changing silicone layer softness and thickness between the two electrodes. A separate ANN real-time calibration model is used for each attached sensor to measure the finger’s forces applied to the object. Equal pressure with small pumps and solenoid valves is applied to two fingers simultaneously. Figure 12b shows the calculated forces with 10 kPa pressure in each calibrated sensor as the radius of the circle. The two sensors show approximately the same force of 2.5 N. The potential challenge of this sensor is when the bending angle is large and affects the sensor’s performance. Dividing the sensor into separate parts and designing some spacers between each part could solve this problem. However, this will require a precise fabrication and molding procedure.

## 5. Conclusions

This work presented a wide area covering tactile sensors for soft robotic applications. The lower layer was made of silicone films embedded in a paper completely covered with conductive ink. The top layer was made of a paper shield employed with conductive ink, which helps measure the electric field changes even for non-conductive objects. A novel, fast, cost-effective fabrication method for this tactile sensor was also proposed in this paper. A large-area 100 × 100 mm soft pad tactile sensing array was presented to study the performance of the proposed sensor. Bringing the object near the surface changes the generated electric field and increases the mutual capacitance. The spatial sensitivity of the sensor was measured, and its capacity to detect simultaneous multi-touch points and to obtain their corresponding contact forces was validated. An experimental setup with different probes was designed to show the consistency and durability of the sensor’s performance. Then, a calibration technique by neural networks was proposed to find the best calibration model. An ATI force sensor was used as a reference for measuring the applied force. An LMBP-ANN training algorithm was executed with a MATLAB program to calculate outputs based on the proposed procedure. The training process of the presented model continued by updating the weight amounts until reaching the highest performance, achieving the minimum MSE. After calibration, the derived models were tested by using a two-fingered soft gripper to grasp a Rubik’s cube, where two of the proposed soft sensors were pushed against this object. The experiment showed that the sensors measured the applied forces and contact points with a good approximation. The proposed sensor covered a large surface area of the gripper, which is very useful for soft robot grippers in detecting several contact points, while in rigid grippers, only the tipping point is important as a contact location. Future works will be primarily needed to improve the sensor’s long-term stability and resolution. These may include efforts to print the electrodes with conductive ink and use resin-coated papers to reduce the resistance and increase the sensitivity.

## Figures and Tables

**Table 1:** Material properties of Ecoflex 00-50.

Specific gravity	1.07 g/cc
Cure time	3 h
Shore hardness	00-50
Tensile strength	315 psi
100% modulus	12 psi
Elongation @ break	980%
Mixing ratio	1A:1B
Color	Translucent
Mixed viscosity	8000 cps

**Table 2:** Comparison of the total cost of our proposed soft tactile/capacitance sensor with previous well-known approaches.

Method	Sensor Body		Electrodes		Fabrication Cost
	Type	Price	Type	Price	
Proposed sensor	Silicone	+	Conductive ink	+	+
Kim et al. [20]	Silicone	+	Liquid metal EGaIn	++++	+++
Cheng et al. [24]	Polydimethylsiloxane (PDMS)	++	Copper wires	+	++
Lipomi et al. [43]	PDMS	++	Carbon nanotube	++	+++
Yao et al. [44]	Silicone	+	Silver nanowire AgNW	++++	+++

**Table 3:** Specifications and parameters of the ANN model.

Training Parameters	Values
Neural network model	Feedforward
Input layer	1
Hidden layer	1
Hidden layer neurons	3
Output layer	1
Training network algorithm	Levenberg–Marquardt back-propagation
Training percentage	70%
Testing percentage	15%
Validation percentage	15%
Transfer function hidden layer	Tan-sigmoid
Transfer function output layer	Pure line
Data division	Random
No. of epochs	51
Validation checks (iterations)	6
Performance	Mean squared error (MSE)

## Data Availability

All data collected during this research is presented in full in this manuscript.
